# Evaluating health technology engagement among family caregivers of patients undergoing hematopoietic cell transplantation

**DOI:** 10.21203/rs.3.rs-427058/v1

**Published:** 2021-05-14

**Authors:** Minakshi Raj, Vibhuti Gupta, Flora Hoodin, Lilian Yahng, Thomas Braun, Sung Won Choi

**Affiliations:** University of Illinois at Urbana-Champaign; Meharry Medical College; Eastern Michigan University; Indiana University Bloomington; University of Michigan School of Public Health; University of Michigan Medicine: University of Michigan Michigan Medicine

**Keywords:** hematopoietic cell transplantation, family caregivers, digital health technology, mHealth

## Abstract

**Purpose:**

Digital health technology-based interventions have the potential to support caregivers in their caregiving responsibilities and in managing their own health and well-being. Designing digital health technologies to support caregivers of patients undergoing hematopoietic cell transplantation requires evaluating their engagement with these technologies. The objective of this study was to examine the association between caregiving characteristics and different types of digital health technologies used.

**Methods:**

We conducted an online cross-sectional, national survey of 948 unpaid family caregivers of patients undergoing hematopoietic cell transplantation.

**Results:**

Almost two-thirds (65.4%) of respondents reported using an app for fitness or step counting, while 41.3% reported using a smartwatch. The average number of apps used was 3.3 (range 0-9). In adjusted models, adult children who were caregivers (OR=5.82, p<0.005) and caregivers of another relative (OR=2.51, p<0.005) were significantly more likely to use a fitness tracker than caregivers of a child. Caregiving for six months or greater was associated with use of fewer apps compared with caregiving for less than six months in adjusted models (OR=0.80, p<0.005). Caregivers of patients receiving an allogeneic transplant used more apps on average than caregivers of patients receiving an autologous transplant, in adjusted (OR=1.36, p<0.005) models.

**Conclusion:**

Digital health technologies may reflect promising avenues for supporting caregivers of patients undergoing HCT. The rapid insurgence of telehealth, propelled by the current COVID-19 pandemic, emphasizes the need for a better understanding of digital health technology for future study design.

## Introduction

The needs of family cancer caregivers are complex, primarily driven by the patients’ illness trajectory [1]. The burdens associated with caregiving are particularly well-established in literature on dementia caregiving with consequences including higher rates of insomnia, depression, chronic and serious illness, and lower likelihood of engaging in preventive health measures [2–7]. With improved cancer survivorship, there may be demands of long-term caregiving and associated health consequences for cancer caregivers that are similar to demands and associated burdens of dementia caregiving. Indeed, patients undergoing hematopoietic cell transplantation (HCT) are considered medically fragile and have unique needs and challenges requiring intense and episodic support from caregivers [8–10]. Thus, HCT caregivers must address the challenging and stressful caregiving needs of some of the most critically ill cancer patients – needs that continue throughout a prolonged hospital stay, followed by close outpatient follow-up over a period of many months [11, 12].

In recent years, digital health technology-based interventions have emerged as a novel platform to support caregivers in their caregiving responsibilities as well as in managing their own health and well-being [13, 14]. However, current interventions have focused primarily on supporting caregivers in their role and responsibility as a caregiver. Interventions that address self-care as a means for caregivers to improve and maintain their own physical, mental, and spiritual health remain in formative stages [15]. For instance, mHealth and wearable sensors, which are increasingly being leveraged to support health care delivery, can help individuals monitor and manage their health and health behaviors and can also be integrated with the electronic health record so that health information collected outside of health care visits can enhance health care supported by the electronic health record [16].

Prior studies have documented demographic predictors of wearables and mHealth apps, such as age (younger), income (higher), education (college), gender (female), and race (white) [17, 18]. However, use of wearables and mHealth applications among caregivers of patients with cancer has not been widely studied to our knowledge [19]. These technologies may support caregivers in health and health behaviors, such as physical activity, which may also be related to how caregivers cope with various daily stressors. For instance, Litzelman and colleagues found that problem-based coping was associated with more physical activity when multiple regression analyses controlled for demographic and caregiving characteristics, such as hours per week of care, participation timepoint, and care recipient age and gender [20]. Assuming caregivers who use mHealth apps and wearables would do so to monitor their physical health and health behaviors and possibly also as a means of coping, it is possible that coping style is also related to use of wearables and mHealth apps.

Based on the premise that digital health technology-based interventions have potential to support family caregivers maintain health behaviors, in the current study we drew upon our National Caregiver Health Survey of caregivers of HCT patients [10, 21] to examine the association between caregiving characteristics and different types of digital health technologies used. Investigation into the use of different types of technologies could thus be important to determine their potential impact on caregivers, and may also inform future digital health technology-mediated intervention design.

## Methods

In this manuscript, we report findings from our National Caregiver Health Survey [10, 21]. which is part of a larger, multi-phase project. The survey was implemented by the Center for Survey Research at Indiana University (LY). The survey itself was developed through cognitive interviews of HCT caregivers using verbal probing and think-aloud approaches [8, 10, 30, 22–29].

### Sampling frames

Sampling frames were from email distribution lists from the National Bone Marrow Transplant Link (nbmtLINK) and Blood and Marrow Transplant Information Network (BMT InfoNet). Both are nonprofit organizations in the U.S. that serve transplant patients and family caregivers. Upon receiving approval from the institutional review board, the nbmtLINK and BMT InfoNet advertised and provided access to the survey in their electronic newsletters and email distribution lists which include volunteers who opt into the lists. In addition, we obtained survey responses through a study brochure containing the URL and QR code that was distributed at BMT InfoNet’s Celebrating a Second Chance at Life Survivorship Symposium in May 2019. All members of the email lists were sampled, but there were other sources of potential error, such as nonresponse and measurement errors. Data were cleaned to eliminate duplicate responses [31].

### Data collection

This survey was administered online using Qualtrics software (Qualtrics XM; www.qualtrics.com) between May 2 and June 30, 2019. Inclusion criteria included being an unpaid family caregiver of an HCT recipient, 18 years of age or older, and able to complete the survey online in English. Respondents received a $20 Amazon gift card upon survey completion. The survey was approximately 16 minutes in duration.

### Measures

#### Digital Health Technology Use:

Our outcomes included three measures of the nature and extent of digital health technology use: use of a fitness tracker (yes/no), use of a smartwatch (yes/no), and total number of health apps used currently (ranging from 0 to 9). Fitness trackers and smartwatches both track physiological parameters, but smartwatches provide additional capabilities (email, call, text).

#### Caregiving characteristics.

The six specific features of caregiving having potential association with digital health technology use were: caregiver type of relationship to care recipient (e.g., parent, adult child, spouse/partner, another relative); whether the caregiver also supported others (yes vs no), care duration (less than six months vs. more than six months), care burden (hours per week: less than 20 vs. 20–40 vs. 40 or more); whether the caregiver lived with the care recipient (yes vs. no); and donor source (autologous vs. allogeneic).

#### Coping:

We used a subset of 16 items from the 28-item Brief COPE, which assesses fourteen different coping strategies including self-distraction, active coping, denial, alcohol and drug use, use of emotional support, use of instrumental support, behavioral disengagement, venting, positive framing, planning use of humor, acceptance, and religion [20, 21]. We used factor analysis to identify a set of four unique coping factors: strategic, emotional, religious, and social support. The mean response to the component items in each factor served as the caregiver’s score for that factor.

#### Caregiver Demographics:

We also incorporated eight caregiver demographics that have been identified in previous studies as being important predictors of digital health technology use (age, sex, race, ethnicity, income, education, marital status, employment status).

### Statistical Methods

All continuous measures were summarized with a sample mean and sample standard deviation, while all categorical measures were summarized with the proportion of the sample in each category. We also tabulated the percentage of missing values for each measure. Before any analyses were done, we used multiple imputation with chained equations (MICE) [32] to impute the missing values for each respondent; all analysis results are based upon ten imputed datasets. We individually assessed the (unadjusted) association of each caregiving factor with the probability of using a fitness tracker and probability of using of smart watch with logistic regression and with the mean number of apps with Poisson regression. We then incorporated all seven caregiver demographics and four coping measures into the regression models to produce an adjusted association of each caregiving factor with each measure of digital health technology use. In all models, statistical significance was defined as a *p*-value less than 0.005 to account for multiple comparisons and limit false positive findings. All analyses were done in the statistical software R (version 4.0.4).

## Results

### Caregiver demographics.

The median age of the survey respondents (n = 948) was 40 years (range 18–89 years) (Table 1). A majority (65.4%) identified as female, married (86.8%), and employed (78.4%). Over three-quarters were White (78.7%) and non-Hispanic (82.6%), and reported at least a college education (70.1%) and an annual household income greater than $50,000 US (65.7%).

### Caregiving characteristics.

About one-third of caregivers were parents to their recipient (32.8%). The rest were adult children (28.9%), spouses/partners (27.1%) or another relative, such as a grandparent, cousin, or friend (11.0%). Most caregivers supported another relative (68%) and resided in the same household as their care recipient (82.9%), with about one-quarter (24%) spending greater than 40 hours per week caregiving. Just over half (52.8%) reported caregiving for more than six months.

### Coping characteristics.

Of the four coping strategies emerging from our factor analysis, the mean score for emotional coping was the lowest (mean = 2.5, SD = 0.7), with lower scores being more adaptive. The mean score for strategic coping was highest (mean = 3.2, SD = 0.4), with higher scores being more adaptive. Table 2 summarizes the four coping strategies reported by respondents.

### Digital Health Technology use.

Almost half (45%) of respondents used an iPhone, while 53% used an Android or Windows phone. The remaining 2% used another type of cellphone. About two-thirds (65.4%) of respondents reported using an app for fitness or step counting, while 41.3% reported using a smartwatch. The average number of apps used was 3.3 (range 0–9).

## Associations between caregiving characteristics and digital health technology use

### Fitness tracker.

In unadjusted models, caregivers who were adult children of their care recipient were significantly more likely to use a fitness tracker compared with caregivers who were parents of their care recipient (OR = 5.80, p < 0.005) (Table 3). Caregivers for six months or greater were significantly less likely than those caregiving for less than six months to use a fitness tracker (OR = 0.40, p < 0.005). Donor source also emerged as an important variable with caregivers of patients undergoing allogeneic transplant more likely to use a fitness tracker than caregivers of patients undergoing autologous transplant (OR = 2.02, p < 0.005). After adjusting for demographic and coping characteristics, caregiver relationship remained a significant predictor of use of fitness tracker with adult children who were caregivers (OR = 5.82, p < 0.005), and caregivers of another relative (OR = 2.51, p< 0.005) being significantly more likely to use a fitness tracker than caregivers of a child.

### Smartwatch.

Unadjusted (OR = 0.28, p < 0.005) and adjusted (OR = 0.46, p < 0.005) models showed that spousal caregivers were significantly less likely to use a smartwatch compared with caregivers who were parents of their care recipient (Table 4). Caregivers caring for others were also more likely than those not caring for others to use a smartwatch in both unadjusted (OR = 2.29, p < 0.005) and adjusted (OR = 1.79, p < 0.005) models. Similar to findings associated with use of a fitness tracker, caregivers of patients undergoing allogeneic transplant were more likely to use a smartwatch, in both unadjusted (OR = 4.63, p < 0.005) and adjusted (OR = 3.05, p < 0.005) models.

### Number of health apps.

Multiple caregiving characteristics were associated with the mean number of apps in both unadjusted and adjusted models (Table 5). On average, caregivers who were children of their recipient used more apps than caregivers who were parents of their children, in both unadjusted (OR = 1.23, p < 0.005) and adjusted (OR = 1.28, p < 0.005) models. Caregivers of patients receiving an allogeneic transplant also used more apps on average than caregivers of patients receiving an autologous transplant, in both unadjusted (OR = 1.89, p < 0.005) and adjusted (OR = 1.36, p < 0.005) models. Additionally, caregiving for six months or greater was associated with use of fewer apps compared with caregiving for less than six months in both unadjusted (OR = 0.66, p < 0.005) and adjusted (OR = 0.80, p < 0.005) models.

## Discussion

In this study, we analyzed responses from 948 caregivers of our National Caregiver Health Survey to assess the relationship between caregiver characteristics and use of three types of digital health technology: fitness tracker, smartwatch, and mobile apps. Prior studies have examined use of fitness trackers, smartwatches, and number of mobile apps among other populations, including the general US public, caregivers of children with cancer, older adults with dementia, and caregivers of older adults [17, 33–36]. However, to our knowledge, these variables (fitness tracker, smartwatches and number of apps) have not been explored in the context of caregivers of patients undergoing HCT. With the rapid insurgence of telehealth, propelled by the current COVID-19 pandemic, having a better understanding of health technology is important for future study design [17, 33, 36, 37].

Herein, two caregiver characteristics emerged in the use of digital health technology: i) caregiver relationship with care recipient and ii) care burden (i.e., hours per week spent caregiving and allogeneic vs. autologous transplant type). Interestingly, compared with parents caregiving for their children as the referent group, spousal caregivers were the least likely group to use a fitness tracker, smartwatch, or mobile apps. It is possible that the other cohorts of caregivers (e.g., parent caregivers, adult child caregivers, other caregivers) were more likely to have younger aged children, often referred to as the “sandwich generation,” based on having dual responsibilities to both a younger and older generation. Further, despite adjusting for coping styles in order to account for possible individual differences, we found no evidence of a relationship between any specific coping approach and use of fitness-related technology. Thus, our study contrasts with prior findings that problem-solving coping was associated with caregiver physical activity [20].

Our data suggest relatively high adoption of digital health technology (e.g., fitness trackers, smartwatches, mobile apps) among HCT caregivers in this national sample. Caregiver use of fitness trackers in our sample (40–65%) was slightly higher compared with digital health technology use among the general adult public, as reported in other studies (20–30%) [17, 38]. There may be possible explanations for higher use of digital health technology (e.g., fitness tracker, smartwatches, mobile apps) among caregivers [35]. Considerable literature has established the time factor associated with caregiver burden as well as the impact on caregivers’ capacity to care for their own health (self-care) [39–41]. It is possible that these technologies enable caregivers to monitor their own health in an accessible way and provide a means to support their well-being. In the current climate of extended use of telehealth during the pandemic, incorporating digital health technology, such as non-invasive, consumer-grade wearables, might give providers a platform for real-time monitoring in the outpatient setting of both caregivers and patients. Given that almost all participants in our sample own a smartphone, providers and health systems could create and implement novel ways to reach a large number of caregivers through mobile apps.

Findings from this study also provide us with a greater understanding of the needs of HCT caregivers who are experiencing greater burden associated with their responsibilities (i.e., differential circumstances based on caregiver type – spouse, parent vs. adult child) and with their care recipient’s needs (i.e., allogeneic vs autologous transplant). Indeed, our study suggests that digital health technology may play a role in supporting family caregivers. Future research should examine the circumstances under which caregivers use different types of digital health technology, the frequency of this use, and caregiver outcomes such as their ability to meet their own lifestyle goals while supporting a care recipient. Further, clinical trials could incorporate consumer technologies to collect physiological and/or health behavior data. Most importantly, interventions to support caregivers should be tailored to “at risk” subgroups based on caregiver burden (i.e., time spent caregiving, donor type) or relationship between caregiver and care recipient. New interventions may with confidence incorporate a fitness tracker for caregivers supporting their parents. Future research should identify the types of technologies that spousal caregivers are more likely to use, in addition to potential barriers to their use before attempts are made to tailor interventions using fitness technologies for this segment of caregivers.

There are some limitations to our study. First, our study was conducted at a cross-sectional time point in each of the caregiver’s transplant journey. Thus, findings may not be generalizable across all caregivers of patients undergoing HCT. Our study may reflect some unavoidable selection bias, such as high proficiency in digital health technology use among participants. Further, respondents may have been able to participate in the study because they may have been receiving caregiving support, thereby enabling them to do so, or they may not have been experiencing as much burden associated with their caregiving responsibilities. The sample was also highly educated and relatively affluent.

## Conclusion

The findings herein support that consumer digital health technologies, including fitness trackers, smartwatches, and mobile apps, may be promising avenues for supporting caregivers of patients undergoing HCT. Our research team is currently conducting a mobile health randomized trial in family caregivers and patients undergoing HCT on the use of the Roadmap app, which is a positive psychology-based intervention. The Roadmap app also passively collects physiological data, including heart rate, steps, and sleep, through Fitbit Charge 3 devices and their API (https://roadmap.study; registered on ClinicalTrials.gov NCT).

## Figures and Tables

**Table 1:** Summary of caregiver demographics and caregiving characteristics.

Demographic	Category	N (%)
Age	≤ 40 years	479 (50.5)
	> 40 years	465 (49.1)
	Not Reported	4 (0.4)
Gender	Male	324 (34.2)
	Female	620 (65.4)
	Not Reported	4 (0.4)
Income	≤$50,000	249 (26.3)
	$50,001-$99,999	373 (39.3)
	≥$100,000	250 (26.4)
	Not Reported	76 (8)
White Race	Yes	746 (78.7)
	No	194 (20.5)
	Not Reported	8 (0.8)
Hispanic Ethnicity	Yes	158 (16.7)
	No	783 (82.6)
	Not Reported	7 (0.7)
Currently Married	Yes	823 (86.8)
	No	120 (12.7)
	Not Reported	5 (0.5)
Employed	Yes	743 (78.4)
	No	197 (20.8)
	Not Reported	8 (0.8)
Education Attained	Some college or less	280 (29.5)
	College degree or more	665 (70.1)
	Not Reported	3 (0.3)
**Characteristic**		
Caregiver Relation to Recipient	Parent	311 (32.8)
	Adult Child	274 (28.9)
	Spouse	257 (27.1)
	Other Relation	104 (11.0)
	Not Reported	2 (0.2)
Transplant Type	Allogeneic	804(84.8)
	Autologous	130 (13.7)
	Not Reported	14 (1.5)
Caring for Others	Yes	644 (67.9)
	No	301 (31.8)
	Not Reported	3 (0.3)
Care Duration	≤ 6 months	443 (46.7)
	> 6 months	501 (52.8)
	Not Reported	4 (0.4)
Weekly burden	≤ 20 hours/week	343 (36.2)
	20–40 hours/week	376 (39.7)
	> 40 hours/week	224 (23.6)
	Not Reported	5 (0.5)
Lives with Recipient	Yes	786 (82.9)
	No	156 (16.5)
	Not Reported	6 (0.6)

**Table 2:** Summary of four coping strategies

Measure	Mean (SD)	% Missing
**Strategic**	3.2 (0.4)	6.5%
**Emotional**	2.5 (0.7)	8.2%
**Religious**	3.0 (0.8)	5.4%
**Social Support**	3.0 (0.7)	5.1%

**Table 3:** Unadjusted and adjusted associations of caregiving characteristics with use of fitness tracker^a^

Caregiving characteristic	Unadjusted		Adjusted for demographics and coping	
	Odds Ratio	95% CI	Odds Ratio	95% CI
**Caregiver relationship to recipient**				
Parent	Ref	Ref	Ref	Ref
Adult child	**5.80**	**[3.89, 8.66]**	**5.82**	**[3.62, 9.36]**
Spouse	0.82	[0.59, 1.15]	0.86	[0.57, 1.30]
Other	1.66	[1.05, 2.62]	2.51	[1.49, 4.21]
**Care for others**				
No	Ref		Ref	
Yes	1.41	[1.07, 1.87]	0.96	(0.67, 1.38)
**Care duration**				
Less than six months	Ref		Ref	
Six months or greater	**0.40**	**(0.30, 0.52)**	**0.46**	**(0.32, 0.66)**
**Care burden**				
Less than 20 hours/week	Ref		Ref	
20–40 hours/week	**1.79**	**(1.32, 2.42)**	1.41	(1.01, 1.98)
40 or more hours/week	0.93	(0.66, 1.30)	0.93	(0.62, 1.39)
**Caregiver lives with care recipient**				
No	Ref		Ref	
Yes	0.84	(0.59, 1.20)	0.73	(0.49, 1.08)
**Donor type**^b^				
Autologous	Ref		Ref	
Allogeneic	**2.02**	**(1.39, 2.95)**	1.41	(0.91, 2.17)

a Boldface indicates p < 0.005

b Autologous = self-donation of hematopoietic stem cells (HSCs); Allogeneic = donation of HSCs by donor other than self (e.g., sibling, relative, unrelated donor)

**Table 4:** Associations of caregiving characteristics with use of smartwatch^b^

Caregiving characteristics	Unadjusted		Adjusted for demographics and coping	
	Odds Ratio	95% CI	Odds Ratio	95% CI
**Caregiver relationship to recipient**				
Parent	Ref	Ref	Ref	Ref
Adult child	1.16	(0.83, 1.60)	1.14	(0.75, 1.73)
Spouse/partner	**0.28**	**(0.19, 0.42)**	**0.46**	**(0.29, 0.72)**
Other	1.06	(0.67, 1.67)	1.10	(0.66, 1.83)
**Care for others**				
No	Ref			
Yes	**2.29**	**(1.70, 3.08)**	**1.79**	**(1.2, 2.67)**
**Care duration**				
Less than six months	Ref			
Six months or greater	**0.58**	**(0.44, 0.76)**	0.70	(0.48, 1.00)
**Care burden**				
Less than 20 hours/week	Ref		Ref	
20–40 hours/week	0.91	(0.68, 1.23)	0.87	(0.62, 1.22)
40 or more hours/week	0.61	(0.43, 0.86)	0.80	(0.52, 1.25)
**Caregiver lives with care recipient**				
No	Ref		Ref	
Yes	1.07	(0.75, 1.52)	1.25	(0.84, 1.86)
**Donor type**^c^				
Autologous	Ref		Ref	
Allogeneic	**4.63**	**(2.72, 7.88)**	**3.05**	**(1.70, 5.47)**

c Boldface indicates p < 0.005

**Table 5:** Unadjusted and adjusted associations of caregiving characteristics with mean number of apps^d^

Caregiving characteristic	Unadjusted		Adjusted for demographics and coping	
	Mean Ratio	95% CI	Mean Ratio	95% CI
**Caregiver relationship to recipient**				
Parent	Ref	Ref	Ref	Ref
Adult child	**1.23**	**(1.13, 1.34)**	**1.28**	**(1.15, 1.42)**
Spouse/partner	**0.56**	**(0.50, 0.63)**	**0.72**	**(0.63, 0.83)**
Other	1.05	(0.93, 1.18)	**1.21**	**(1.07, 1.37)**
**Care for others**				
No	Ref			
Yes	**1.63**	**(1.49, 1.79)**	**1.29**	**(1.16, 1.44)**
**Care duration**				
Less than six months	Ref			
Six months or greater	**0.66**	**(0.61, 0.71)**	**0.80**	**(0.73, 0.88)**
**Care burden**				
Less than 20 hours/week	Ref		Ref	
20–40 hours/week	**1.14**	**(1.05, 1.24)**	1.11	(1.02, 1.21)
40 or more hours/week	**0.81**	**(0.73, 0.91)**	0.98	(0.87, 1.11)
**Caregiver lives with care recipient**				
No	Ref		Ref	
Yes	0.95	(0.87, 1.05)	0.97	(0.88, 1.07)
**Donor type**				
Autologous	Ref		Ref	
Allogeneic	**1.89**	**(1.60, 2.23)**	**1.36**	**(1.15, 1.60)**

d Boldface indicates p < 0.005
